# Surgery

**Published:** 1906-03-10

**Authors:** 


### SURGERY.

**Surgery of Cerebral Tumours.**—Unfortunately, from their very nature and position, the majority of cerebral tumours do not admit of radical removal. But nevertheless operative treatment can often afford definite palliative relief of the otherwise intolerable symptoms. Walton,[1] from a careful pathological and clinical examination of 374 cases, including 171 autopsies, comes to the conclusion that 7.5 per cent. are operable, 79.4 per cent. are inoperable, and 13.1 per cent. are doubtful. The *operable* class include those tumours which have most defined limits and are situated in accessible regions. They are mostly endotheliomata of dural origin. The *inoperable* class include all tumours involving the deep parts of the brain, and also multiple or metastatic growths. The *doubtful* class includes gliomata, unencapsuled sarcomata, and cysts. Attention is drawn to the number of cases where operable endotheliomata were found in the mid line or under the frontal lobe in which no localising signs existed. Cushing [2] strongly advocates an attempt being made to relieve the agonising pain and other symptoms of inoperable tumours of the brain. He points out that these symptoms are due mainly to pressure caused by a growing foreign body in the rigid cranium. If a large area of the skull be removed so as to "decompress" the brain, a hernia cerebri will develop. If this is done, however, over the "silent" areas of the cerebral cortex, no very great harm will result. He finds the best region in which to operate is the temporal fossa under the temporal muscle, which affords a certain degree of protection to the otherwise exposed brain. He relates 15 cases in which such operations have been performed with very gratifying results.

**Tumours involving the Cauda Equina.**—These very rare cases have a fairly typical history and symptomatology. Three situations of the growth are possible, and can often be differentiated from one another. First, in the conus medullaris; second, in the dura pressing on the nerves of the cauda equina; and third, in the sacral bones. When the conus is involved the case pursues a rapid course. Motor paralysis is even more marked than sensory, and the centres for the bladder and rectum are early involved. Schmoll [3] relates a typical case of the second variety. The patient was a man of 42, who for three and a half years had suffered from paroxysms of double sciatica. The sciatic nerves were so tender that he could hardly sit or lie on his back. A very tender area, over which was some indefinite swelling, was present over the third sacral vertebra. The pain was particularly severe after defæcation. An opening was made into the canal of the sacral vertebra over the point of swelling, and a tumour 6 c.m. long by 2 c.m. wide was easily shelled out from beneath the dura mater. It was a gliosarcoma of benign type. Complete relief from pain was afforded by the operation, but the patient died of septicæmia four days afterwards. Verdi [4] describes a case where the tumour was of a bony nature. The patient was a man of 44. He suffered from paroxysms of pain in the hypogastrium. Later he suffered from a fixed pain radiating from the second lumbar vertebra behind to the supra-pubic region in front. In addition he had some recent ocular paralysis. No motor or sensory paralysis was present. The laminæ of the first, second, and third lumbar vertebræ were removed, and growing from the deep surface of the last-named bone was a bony tumour which measured 1 inch by ⅝ inch, and which almost occluded the canal. Its removal gave complete relief to the symtoms.

*Surgical Repair of Injured Nerves.*—Horsley [5] relates the case of a patient in whom, as the result of an accident, the upper part of the median nerve had been destroyed for 2½ inches, and the musculo spiral nerve was also injured in the lower part of the arm, producing paralysis of all the muscles of the forearm except those supplied by the ulnar nerve. Three months later the median nerve was implanted laterally into the ulnar, and 14 months later the masculo-spiral nerve was implanted into the median. Ten months after the second operation motion and sensation had been almost entirely restored to the forearm.

**Spinal Surgery.**—A valuable paper dealing with the subject of fractures of the spine, and based upon the records of 224 cases is given by Burrell.[6] Of the whole number only 35 per cent. recovered, and a third of these were "useless" in after-life. But whereas in the period 1864 to 1887, when expectant treatment alone had been used the recoveries were only 22 per cent., and the "useful" out of these 50 per cent.; in later years recoveries were 62 per cent., and the useful proportion 76. But this is partly accounted for by the fact that diagnosis is more accurate nowadays, and therefore the later lists contain a larger proportion of trivial cases. It is perfectly clear that the mortality and "uselessness" of recovered cases both depend upon the degree of injury of the spinal cord itself. Although this injury may be the result of crushing or tearing, it may, on the other hand, be caused by mere pressure—*e.g.*, by bone, blood, or exudate. If, however, pressure upon the cord is allowed to remain for more than a few hours irremediable damage to the cord may occur. In all cases presenting evidences of injury to the cord, therefore, unless it is perfectly clear that the cord is irreparably damaged, an open operation should be performed—*i.e.*, laminectomy, to ascertain the condition of the cord and to remove pressure from it. In certain cases where the cord is not injured, but is liable to injury from displacement of the fragments of a vertebra, rectification of the deformity and fixation of the spine may be used. The treatment of Pott's disease of the spine is a most anxious and trying process, and it has been proposed to shorten this treatment by various heroic procedures—namely, forcible straightening of the spine, laminectomy, or excision of carious vertebræ. Willard [7] summarises his opinions on this subject thus:—Long continued hyperextension and fixation amid healthy surroundings must remain the best treatment for the majority of cases. Forcible gradual straightening is a valuable proceeding for the reduction of deformity. When reduced the spine must be fixed in plaster of paris. Forcible immediate straightening is always unjustifiable. It may lead to sudden death or to a reawakening to activity of dormant tubercular foci. Laminectomy for paraplegia is only justifiable when rest and extension have been tried for one or two years. The operation has a mortality of 25 per cent. from immediate shock, 50 per cent. die within 12 months, and of those that survive 15 per cent. show no improvement. The uncertainty and danger of removing carious bodies of vertebræ excludes this operation from practical surgery. Wiring together spinous processes so as to prevent kyphosis may be useful, but has not been tried sufficiently to give an opinion. The spinal cord is regarded as a tissue practically incapable of repair from the results of surgical injuries. But one case has been recorded by Stewart and Harte in which the cord, which had been completely severed, was sewn together three hours after the injury. And a considerable degree of motion and sensation below the lesion showed that a large measure of repair must have occurred. Fowler [8] relates a similar case. The patient was a boy of 18, who had been shot by a bullet ten days previously. An incision six inches in length was made over the spines of the vertebræ, the middle being over the eleventh dorsal spine. The bullet, embedded in blood-clot, was found between the severed ends of the cord. After removal of clot and bullet the ends of the cord were sewn together with fine catgut suture. But 26 weeks after there were no signs of return of motion or sensation below the wound except for one area of skin on the outer side of the right thigh, in which sensation was present. This clearly shows that the first essential to success in operating on the spinal cord is to operate at once before degenerative changes have had time to become established.

*Tumours of the Spinal Cord and its Membranes* are of rare occurrence, but the results of surgical treatment are distinctly encouraging. Harte [9] has collected and analysed 92 cases which were operated upon. Of these 49 recovered, and 29 were cured; 37 out of the number were said to be sarcomata, but in the case of earlier records the possibility of some of these being endotheliomata must be borne in mind. At any rate the tendency to recurrence in these cases does not seem to be great. As regards the relationship to the dura mater, 50 were extradural and 36 intradural. The nature of the growth and its relation to the dura cannot be diagnosed before operation. The upper dorsal region was affected in 33 cases, the lower dorsal in 24, the lumbo-sacral in 14, and the cervical in 11. The average duration of symptoms was two years and three months before the operation. In cases which recover the improvement in symptoms is gradual. But pain is generally relieved from the first, then sensation returns, and voluntary motion is recovered latest.

*Lumbar Puncture* must be regarded as having a very important bearing, not only on the diagnosis, but also on the treatment, of cerebral injuries and disease. By withdrawing some of the cerebro-spinal fluid a rapid relief is given to intracranial pressure, and possibly toxic products are removed. Quénu [10] alludes to seven cases of fractured cranial base which were systematically treated by repeated lumbar puncture, with very good results. In one case, where the posterior fossa of the skull was involved, lumbar puncture was employed eight times, and the patient was discharged cured at the end of three weeks. A very distinct remission of the symptoms, especially the coma, was seen after each puncture.

*The Present Position of Spinal Anæsthesia.*—After many hundreds of cases of intraspinal injection of cocaine had been reported, it was agreed that, although in most cases the anæsthesia from the umbilicus downwards was absolute, yet in so large a number of cases did alarming or fatal symptoms arise that the method fell into disrepute, and in this country has never been largely adopted. Bier,[11] at the German Surgical Congress, introduced the subject, and pointed out that two great improvements have been made in the method which promise to make it a great success. These are the addition of some preparation of the suprarenal gland to the cocaine solution; and secondly, the substitution for cocaine of one of its newer modifications. Of the latter stovain and tropa-cocaine are the most important. Stovain is used in doses of .06 to .04 milligram. Tropa-cocaine in doses of .04 to .07

milligram. Over eleven hundred cases in which these modifications of the method had been used were reported by various speakers, and all agreed that the alarming collapse seen in the older method is never encountered. It is ineffectual in about 5 per cent. of the cases, and is most useful in the aged and decrepit, who take general anæsthetics badly.

[1] Glas. Med. Jour., Dec., 1905. [2] Med. Record, Nov. 18, 1905. [3] Amer. Jour. Med. Sc., Jan., 1906. [4] Med. Record, Jan. 27, 1906. [5] Med. Record, Jan. 6, 1906. [6] Annals of Sur., 1905. [7] Ibid. [8] Ibid., Oct., 1905. [9] Ibid., p. 524. [10] Brit. Med. Jour., Epit., Dec. 9, 1905. [11] Annals of Sur. Dec., 1905.

*(To be continued.)*

